# Red Cross League's Programme

**Published:** 1920-02-28

**Authors:** 


### Red Cross League's Programme.

The first conference of the General Council of the League of Red Cross Societies will open on March 2 in Geneva, and will last eight days. Mr. Henry P. Davison, Chairman of the American Red Cross, who is also Chairman of the Council, will preside.

*The Times* says it is expected that representatives will attend from every country that has entered into the League, which means practically every country in the world outside the late enemy Powers. In all, thirty Red Cross Societies are members of the League, and have been invited. The British Red Cross delegates will be the Hon. Sir Arthur Stanley (Chairman), Sir Arthur Lawley, Sir Robert Philip, and Dr. F. Kay Menzies. For the purposes of the conference Canada, Australia, New Zealand, South Africa, and India will be entitled to send representatives separately as nations.

Two main topics for the programme of the conference are proposed. The first relates to the improvement of public health and the prevention of disease, according to the programme outlined by the Cannes Conference in April, 1919, and the scope it affords for discussion is indicated by the number of departments which have been or are being established in the General Medical Office of the League. They include the following: Medical information and publication, child welfare, nursing, miscellaneous communicable diseases, laboratory, library, museum, sanitary engineering, malaria, vital statistics, tuberculosis, industrial hygiene, and venereal diseases.

The second general topic of the meeting will be the consideration of the programme of service in peace time, of each national Red Cross Society, and the most effective means of extending its membership and resources.

